# pH- and acoustic-responsive platforms based on perfluoropentane-loaded protein nanoparticles for ovarian tumor-targeted ultrasound imaging and therapy

**DOI:** 10.1186/s11671-020-3252-z

**Published:** 2020-02-03

**Authors:** Jianping Li, Hong Ji, Yong Jing, Shiguang Wang

**Affiliations:** 1 Department of Geriatric Medicine, Sichuan Academy of Medical Sciences and Sichuan Provincial People’s Hospital, Chengdu, 610041 Sichuan China; 2 Department of Imaging, Eastern Hospital of Sichuan Academy of Medical Sciences and Sichuan Provincial People’s Hospital, Chengdu, 610000 Sichuan China

**Keywords:** Phase-transformation, Acoustic droplet vaporization, Ferittin, Ultrasound theranostics, Low-intensity focused ultrasound

## Abstract

In this study, we developed a multifunctional ultrasound (US) therapeutic agent that encapsulates perfluoropentane (PFP) into ferritin (FRT) and conjugates the tumor-targeting molecule folic acid (FA) (FA-FRT-PFP). The prepared FA-FRT-PFP had an average particle diameter of 42.8 ± 2.5 nm, a zeta potential of − 41.1 ± 1.7 mV and shows good stability in physiological solution and temperatures. FRT is a pH-sensitive cage protein that, at pH 5.0, disassembles to form pores that can load PFP. The adjustment to neutral pH closes the pores and encapsulates the PFP inside the FRT to form nanoparticles. At pH 5.0, 3 min of low-intensity focused ultrasound (LIFU, 2 W/cm^2^) significantly enhanced the US signal of FA-FRT-PFP through the acoustic droplet vaporization (ADV) effect. Under identical conditions, 4 min of LIFU irradiation caused the bubbles generated by FA-FRT-PFP to break. FA-FRT-PFP could be efficiently targeted into ovarian cancer cells and significantly enhanced the US contrast of FA-FRT-PFP after 3 min of LIFU irradiation. After 4 min of LIFU irradiation, cell viability significantly decreased due to necrosis, likely due to the FA-FRT-PFP mediated release of PFP in the acidic environment of lysosomes after entering the tumor cells. PFP is then transformed into bubbles that burst under LIFU irradiation, forming physical shock waves that lead to the destruction of the cell structure and necrosis, achieving tumor treatment. Taken together, this demonstrates that FA-FRT-PFP is both a novel and promising US theranostics agent for future clinic application.

## Introduction

Ovarian cancer is one of the most lethal female cancers globally [1–3]. The treatment of ovarian cancer remains clinically dependent on radiation therapy, chemotherapy, surgery, and other ancillary therapies [4–6]. However, shortcomings in these strategies continue to hinder improvements in the survival rates of patients. To overcome these shortcomings, more effective and safer diagnostic methods are required. In recent years, the integration of imaging and therapeutics into a single treatment platform has emerged as a hot topic [7–9].

Ultrasound (US) technology, due to its non-invasive, non-radiative, low-cost, and specific characteristics, is widely used in clinical diagnosis and tumor treatments [10–12]. In recent decades, an acoustic response platform (ARP) with US imaging and therapeutic capabilities has been developed for effective tumor diagnosis and treatment [13–16]. ARP mainly includes microbubbles or nanobubbles (MB or NB) and nanoparticle (NP) based platforms [16, 17]. Amongst these ARPs, MB or NB show good acoustic response capabilities, but due to their large size, in vivo tissue penetration is weak and sample stability is poor. NP displays stronger tissue permeability and a longer pharmacokinetic cycle due to its small size. However, the acoustic response capabilities of NPs are limited. It is therefore urgent to design ARPs of small size, with high stability and strong responsiveness.

Liquid fluorocarbons undergo liquid phase gas phase transition in response to external stimulation [18, 19]. Natalya and colleagues developed a nano-emulsion containing liquid fluorocarbon that produced acoustic droplet vaporization (ADV) effects under low-intensity focused ultrasound (LIFU) [20–22]. The emulsions formed bubbles and simultaneously triggered drug release, thereby enabling tumor treatment. However, a layer of sealing material wrapped around the liquid fluorocarbon led to an increase in the intensity and time of the ultrasound required for tumor treatment, resulting in damage to the surrounding healthy tissue. Further optimization of the liquid fluorocarbon carrier is therefore on demand.

In this study, we developed a ferritin (FRT) nanoparticle loaded with liquid fluorocarbon perfluoropentane (PFP) which was further combined with the tumor target molecule folic acid (FA) to form FA-FRT-PFP. Ferritin is a highly biocompatible endogenous nanocage protein that displays pH sensitivity [23–25]. The protein can disassemble in an acidic environment and can be reassembled in an alkaline environment. This permits the controlled loading and release of ferritin in response to pH changes [26–28]. PFP is a frequently used phase-transformation material that shows a boiling temperature of 29 ^o^C. The low boiling temperature facilitated the easy phase-transformation by LIFU which was safer than HIFU. The PFP was loaded into ferritin and the resultant FA-FRT-PFP had a diameter of ~ 47.3 ± 2.8 nm with high stability at physiological solutions and temperatures. According to the results, FA-FRT-PFP had the following advantages: (1) PFP could be easily loaded into FRT by changing the pH from acidic to neutral; (2) under low-intensity focused ultrasound (LIFU, 2 W/cm^2^, 3 min) and pH = 5.0, FA-FRT-PFP releases PFP and undergoes phase changes to enhance US imaging signals through acoustic droplet vaporization (ADV); 3) Under long-term LIFU irradiation (4 min) and pH = 5.0, the PFP released from FA-FRT-PFP exploded and produced a physical shock wave that could effectively destroy tumor cells through necrosis. To our knowledge, this is the first example of PFP loading into FRT as a tumor US theranostics. These results indicate that FA-FRT-PFP + LIFU is a new strategy for integrated tumor diagnosis and treatment.

## Materials and Methods

### Materials

Agarose gel, ferritin (FRT), folic acid (FA), and perfluoropentane (C_3_F_8_, PFP) were purchased from Sigma (St. Louis, MO, USA). NH_2_-PEG_2000_-FA and NH_2_-PEG_2000_-COOH were supplied by Xi’an Ruixi Biotechnology Co., Ltd. (China). 1-ethyl-3-(3-dimethylaminopropyl) carbodiimide (EDC) and N-hydroxysuccinimide (NHS) were bought from Thermo Fisher Scientific (Waltham, MA, USA). Cell counting kit-8 (CCK-8) were obtained from Dojindo Laboratories (Kumamoto, Japan). Dulbecco’s modified Eagle’s medium (DMEM), phosphate-buffered saline (PBS), penicillin–streptomycin, trypsin-EDTA, and fetal bovine serum (FBS) were purchased from Gibco (Grand Island, NY, USA).

### Cell Culture

Human ovarian cancer cell line SK-OV-3 was provided by the Shanghai Institute of Cell Biology, Chinese Academy of Sciences. Normal ovarian epithelial cells HUM-CELL-0088 were obtained from PriCells, Wuhan, China. The cells were cultured in DMEM media containing 10% fetal bovine serum and 1% penicillin-streptomycin solution. The cells were maintained in an incubator with a humidified atmosphere containing 5% CO_2_ at 37° C.

### Preparation of FA-FRT-PFP

Firstly, target molecule FA was conjugated with FRT through the esterification reaction [29]. In brief, NH_2_-PEG_2000_-FA (10 mg) and FRT solution were mixed with the presence of EDC (5 mg/mL) and NHS (20 mg/mL). After 3 h reaction at room temperature with slight stirring, the mixture was purified through dialysis against distilled water (MW cut-off = 1.2 kDa) for 24 h. The resulted solution was FA-FRT nanoparticles. Secondly, the loading of PFP into the cavity of FRT was prepared with the process of denaturation and renaturation of protein [30]. Briefly, FA-FRT was diluted into 5 mL PBS to be 2 mg/mL and adjusted to pH = 5.0 with 30 min slight stirring at room temperature to ensure complete dissociation of the FRT. After that, 500 μl PFP was added into the solution in an ice bath and was subjected to pulse sonication with 5 s on and 5 s pause for a total of 5 min at 80 W in ice bath. After the sonication, the pH value of the mixture was adjusted to neutral (pH = 7.4). As PFP was oil state and unsolvable in water, the excessive PFP was easily removed by liquid separation to be FA-FRT-PFP. FRT-PFP was prepared with a similar method except for changing NH_2_-PEG_2000_-FA into NH_2_-PEG_2000_-COOH.

### Characterization of FA-FRT-PFP

The sizes and zeta potentials of the nanoparticles were tested by a Zeta Sizer (Malvern, NanoZS, UK). The morphology of FA-FRT-PFP was observed by transmission electronic microscopy (TEM, Hitachi, Japan) and inverted fluorescence microscopy (Olympus, Japan). The chemical structure of the nanoparticles was detected by a Fourier transform infrared spectroscopy (Bruker Tensor 27, Bruker Optik, Ettlingen, Germany). The cellular uptake of the FA-FRT-PFP was detected by confocal laser scanning microscopy (LEXT OLS4100, Olympus, Japan). The FITC/PI double-staining cells were analyzed by flow cytometry (BD, Franklin Lakes, NJ, USA).

### The Performance of pH and Ultrasound Responsive Gas-Generating

FA-FRT-PFP in pH = 5.0 and 7.5 condition were irradiated with different acoustic intensity of LIFU (1.5 and 2.0 W/cm^2^) and for different total of time (1, 2, 3, and 4 min) in the agarose gel model, then US images were observed by US equipment (Esaote Mylab 90, Italy) with frequency of 5–12 MHz and mechanical index (MI) of 0.06. After that, the appearance of FA-FRT-PFP was observed by light microscopy in time. The US intensity of the region of interest in US image was analyzed by ImageJ software.

### Cellular Uptake

Briefly, 1 mg of FITC was dissolved in 1 mL of DMSO and then mixed with FRT-PFP and FA-FRT-PFP for 30 min with gentle agitation. Then, the mixed solution was dialyzed overnight in deionized water to remove free FITC and DMSO to obtain FITC-labeled nanoparticles. Next, SK-OV-3 and normal ovarian cells (HUM-CELL-0088) cells were cultured in the medium for 24 h, and then free FITC, FITC-labeled FRT-PFP and FA-FRT-PFP were co-incubated with SK-OV-3 cells for 5 h. In addition, FITC-labeled FA-FRT-PFP was incubated with FA-pretreated SK-OV-3 cells for 5 h. The cells were washed 3 times with PBS and fixed with 4% paraformaldehyde and stained with 0.2 mg/mL of DAPI solution for 10 min. Cells were imaged by confocal laser scanning microscopy to capture fluorescent images. The fluorescence signal was quantitated by a flow cytometer.

### Ultrasound Imaging In Vitro

SK-OV-3 cells were cultured for 24 h in FA-free medium, and then PBS, FRT-PFP and FA-FRT-PFP, and FA-FRT-PFP + FA were incubated with the SK-OV-3 cells for 6 h. The cells were collected and mixed with agarose gel, and then, the cells were irradiated with or without LIFU for 3 min (1 s on and 1 s pause for a total of 3 min; 1 MHz; 2.0 W/cm^2^). At last, US images were captured.

### In Vitro Anticancer Efficacy

SK-OV-3 cells were seeded in a 96-well plate at the density of 1 × 10^4^ cells/well, and allowed to attach for 24 h. For the cytotoxicity of FA-FRT, various concentrations (0, 20, 40, 100, 200, and 500 μg/mL) of FA-FRT were cultured with SK-OV-3 cells. After 24 h treatment, the cells were treated by DMEM media containing 10% CCK-8 for 20 min in an incubator. The absorbance of the cells at 450 nm wavelength was detected by a Multimode Plate Reader (Thermo Scientific) to characterize the cell viability. In addition, the SK-OV-3 cells and normal ovarian cells (HUM-CELL-0088) were treated PBS, FRT-PFP, and FA-FRT-PFP and FA-FRT-PFP + FA for 3 h, and then were irradiated by LIFU (1 s with a 1 s pause for a total of 4 min; 1 MHz; 2.0 W/cm^2^). The treated cells were incubated for another 21 h. As a control, without LIFU, the cells were treated PBS, FRT-PFP, and FA-FRT-PFP and FA-FRT-PFP + FA for 24 h. After that, the viability of the treated cells was detected by CCK-8 assay.

### Cell Death Analysis

To assess the type of cell death, SK-OV-3 cells were seeded in 6-well plates at a density of 5 × 10^4^ cells/mL. After 24 h, the cells were treated with PBS, FRT-PFP, and FA-FRT-PFP and FA-FRT-PFP + FA for 3 h, then irradiated with LIFU (1 s, paused for 1 s for a total of 4 min; 1 MHz; 2.0 W/cm^2^). The treated cells were incubated for an additional 23 h. As a control, cells were treated with PBS, FRT-PFP, and FA-FRT-PFP and FA-FRT-PFP + FA for 24 h in the absence of LIFU. The cells were trypsinized, centrifuged at 1500×*g* for 3 min, washed 3 times with ice-cold PBS, and then resuspended in 200 mL of binding buffer. Thereafter, 5 μL of annexin V-FITC and 10 μL of PI were added and incubated with the cells for 15 min in the dark. The stained cells were analyzed using a flow cytometer.

### Western Blotting

The western blotting was conducted according to previous literature. The cells were treated with 40 μg/mL of PBS (control), FRT-PFP, FA-FRT-PFP + FA, and FA-FRT-PFP combined with or without LIFU irradiation (2.0 W/cm^2^, 4 min) and further 21 h incubation. And then the treated cells were lysed in RIPA buffer (Sigma). Fifty micrograms of each protein with Laemmli sample buffer were boiled for 5 min and subjected to SDS–PAGE. The proteins were transferred onto PVDF membrane (Bio-Rad Laboratories) using semidry Trans-Blot (Bio-Rad Laboratories). Blots were first incubated in TBS blocking buffer containing either 2% milk or 2% BSA (for phospho-specific antibodies) for 1 h at room temperature and then with the respective primary antibodies diluted in TBST (containing 0.1% Tween20 and 2% BSA) overnight at 4 °C. Subsequently, blots were washed and incubated with appropriate secondary antibodies (Santa Cruz) in TBST and detected using SuperSignal West Pico Chemiluminescent Substrate (Thermo).

### Statistical Analysis

All data are presented as mean ± standard deviation. Statistical analyses were performed using Student's t-test. *P < 0.05, **P < 0.01 were considered statistically significant.

## Results and Discussion

### Preparation and Characterization of FA-FRT-PFP

**Fig. 1:**
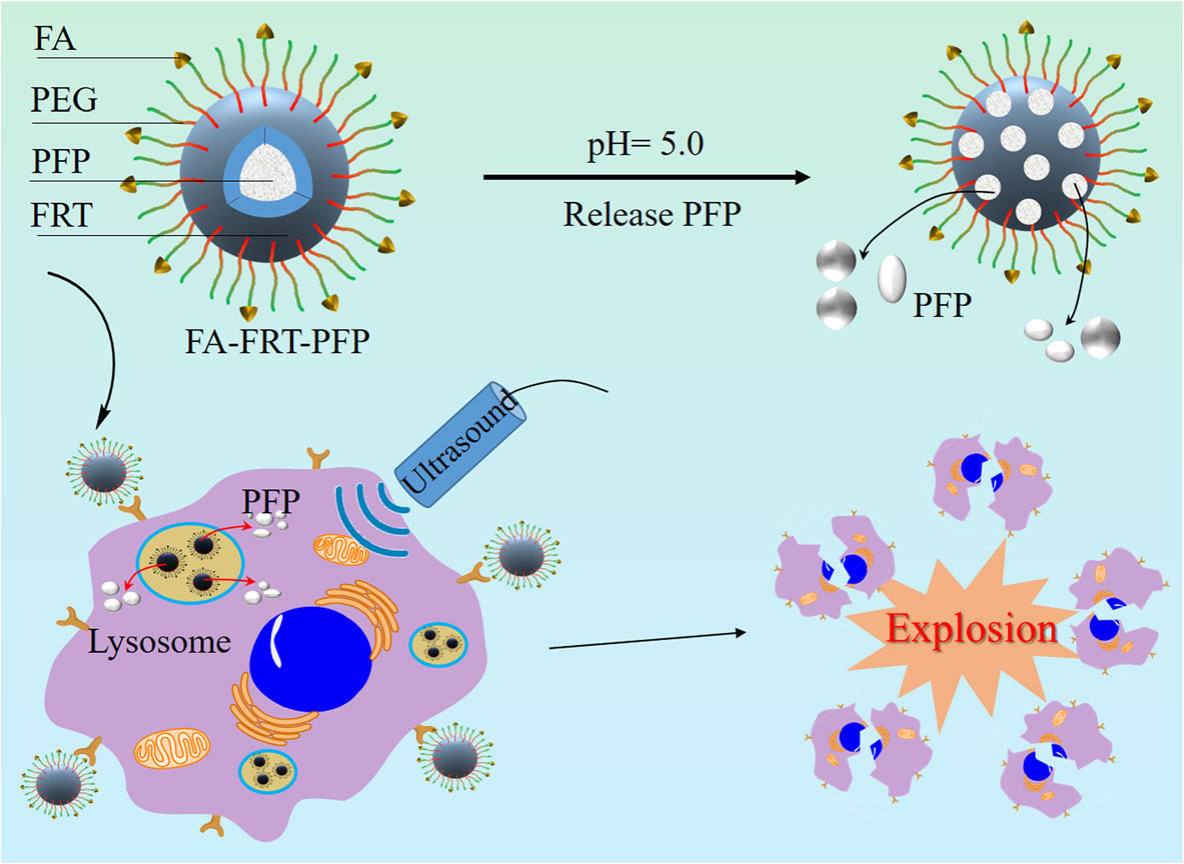
Schematic representation of the synthesis of FA-FRT-PFP and its application in tumor theranostics

**Fig. 2:**
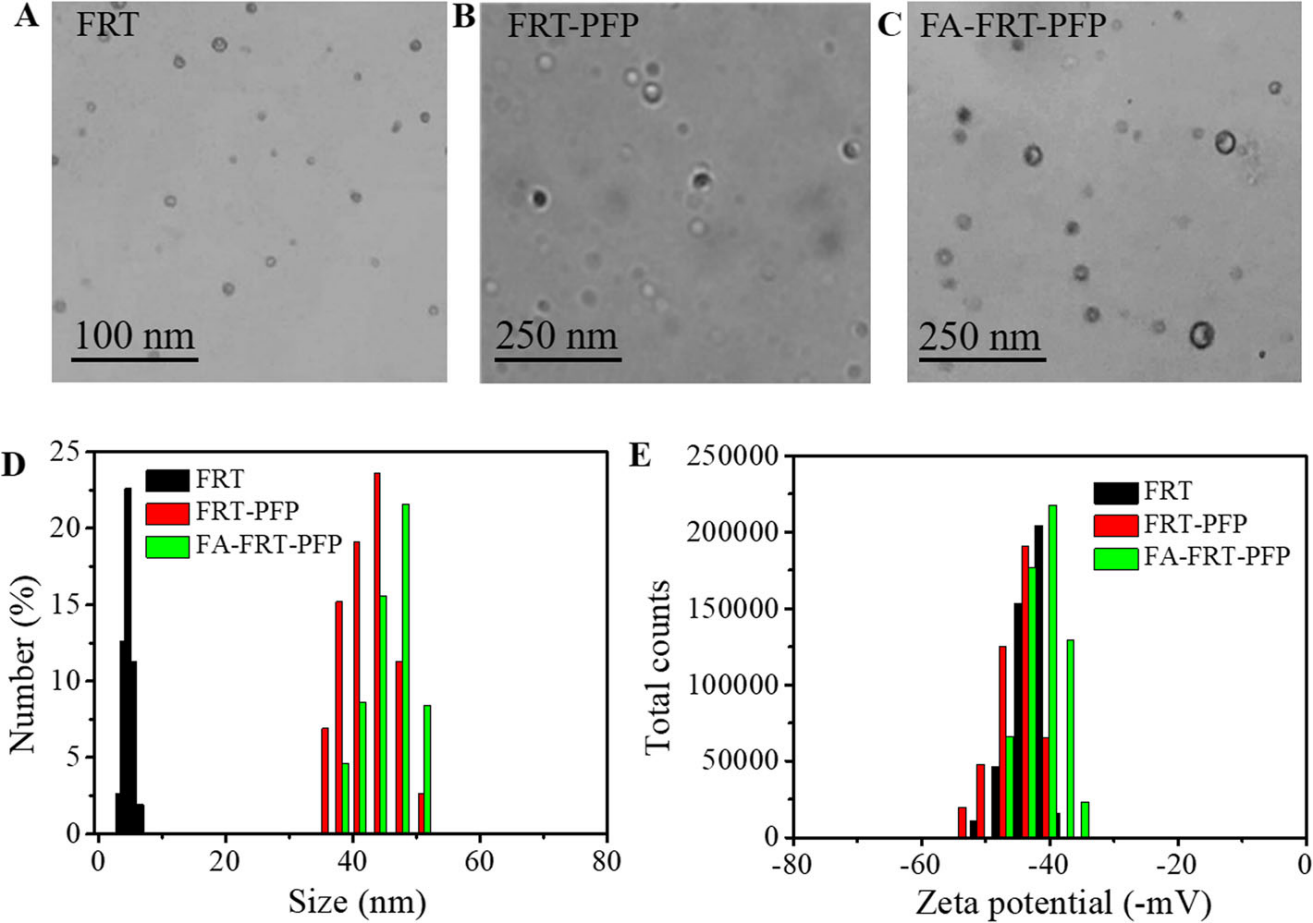
**a**–**c** TEM images of FRT, FRT-PFP, and FA-FRT-PFP. **d**, **e** The size and zeta potential distribution of FRT, FRT-PFP, and FA-FRT-PFP

**Fig. 3:**
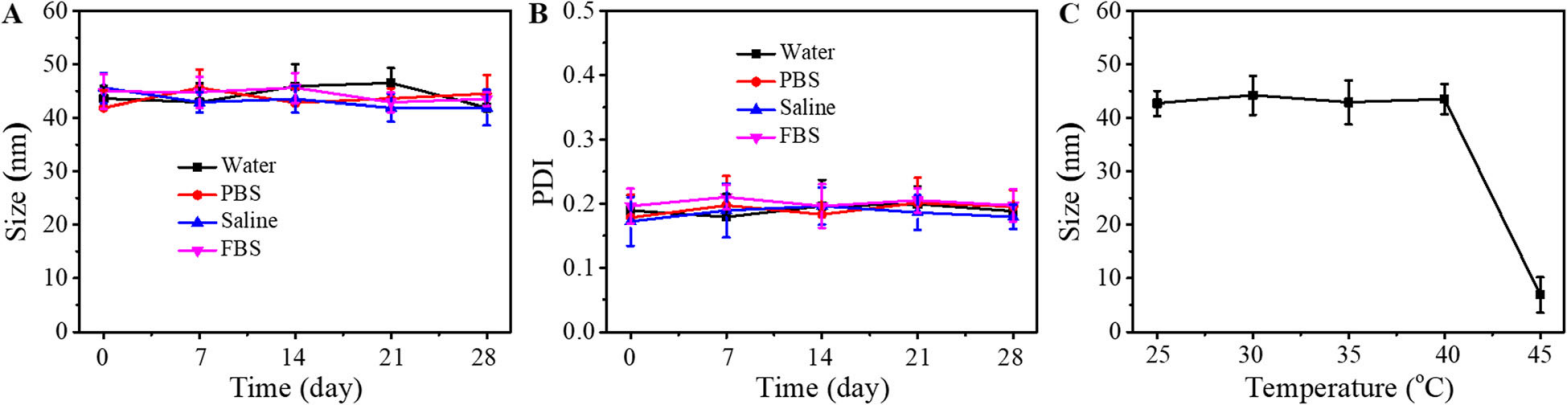
**a**, **b** Size and PDI change of FA-FRT-PFP in water, PBS, saline, and FBS over 28 days. **c** The size change of FA-FRT-PFP at various temperature from 25 ^o^C to 45 ^o^C

FA-FRT-PFP was synthesized and used for tumor therapy (Fig. 1). Phase-transformation droplets were loaded into FRT through the pH-induced reversible disassembly and reassembly of FRT. The PFP with LIFU-induced acoustic droplet vaporization (ADV) was delivered into cells and acted as an ultrasound imaging agent. Figure 2a–c shows TEM images of FRT, FRT-PFP, and FA-FRT-PFP, which were of spherical morphology. The mean particle sizes of FRT, FRT-PFP, and FA-FRT-PFP were 6.9 ± 0.3 nm, 43.8 ± 1.6 nm, and 47.3 ± 2.8 nm, respectively, (Fig. 2d) demonstrating that PFP loading and FA conjugation enhanced the size of FRT. The mean zeta potential of FRT and FA-FRT-PFP were − 43.2 ± 3.1 mV, − 46.9 ± 2.2 mV, and − 41.5 ± 2.7 mV, respectively (Fig. 2e). Moreover, the conjugation of FA with FRT was characterized by FT-IR as shown in Additional file 1: Figure S1. The FT-IR spectrum displayed absorption bands at ~ 1110 cm^−1^ may correspond to the vibrational C–O–C ether linkage of PEG; absorption peaks at ~ 1601 cm^−1^ and ~ 1710 cm^−1^ belong to vibrational C=O bonds from PEG and FRT as well as from –COOH and –CONH_2_ of FA and FRT; the bands at ~ 2820 cm^−1^, ~ 2920 cm^−1^, and ~ 2950 cm^−1^ correspond to asymmetric and symmetric C–H stretch vibrations of –CH_2_ of FA and PEG; absorption peaks at ~ 1440 cm^−1^ are associated with the phenyl ring of FA. The result confirms the successful conjugation of FA with FRT. Over the 28 days storage period, the size and PDI of FA-FRT-PFP in water, PBS, saline, and FBS remained no significant change (Fig. 3a, b), indicating that FA-FRT-PFP shows excellent stability. Figure 3c shows the size change of FA-FRT-PFP at various temperatures ranging from 25 ^o^C to 45 ^o^C. The size of FA-FRT-PFP was almost unchanged when the temperature was reduced to 25–40 °C, indicating that FA-FRT-PFP shows high stability below 40 ^o^C. When the temperature reached 45 °C, the size of FA-FRT-PFP ranged from 42.1 nm to 6 nm, likely due to the PFP in FA-FRT-PFP undergoing severe gasification, leading to rupture of the nanoparticles. The gasification temperature of FA-FRT-PFP increased from 29 ^o^C (gasification temperature of PFP under normal pressure) to 45 ^o^C, likely due to the coverage of the FRT shell [21, 31–33].

### pH and LIFU Responsive US Enhancement

**Fig. 4:**
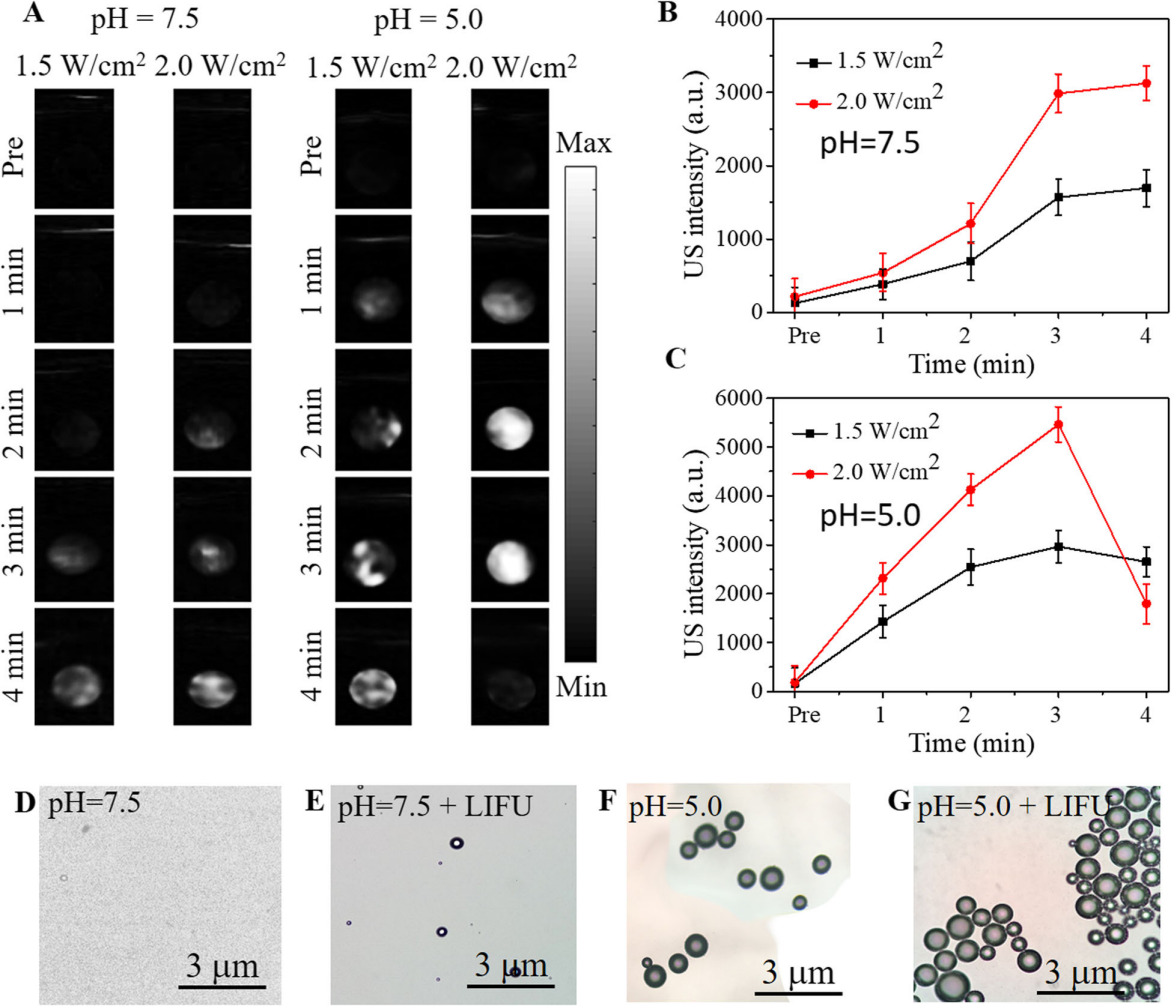
**a** US images of FA-FRT-PFP mixed with agarose gel at different pH values and irradiated by different of LIFU power intensity. **b**, **c** The corresponding statistical data of US signal in **a**. **d**–**g** The optical microscopy images of FA-FRT-PFP mixed with agarose gel at different pH values and irradiated by 2.0 W/cm^2^ for 3 min

US phantom experiments of FA-FRT-PFP were performed at different pH values and LIFU power intensity. As shown in Fig. 4a, in the absence of LIFU irradiation (pre), the US signal was weak in all groups. At pH7.5 after 3 min of LIFU irradiation, FA-FRT-PFP showed a low US signal both at 1.5 and 2.0 W/cm^2^, while at pH = 5.0 under the same LIFU irradiation time, FA-FRT-PFP had a stronger US signal intensity. This demonstrated that the FA-FRT-PFP showed time- and acoustic intensity-dependent ADV effects. This was because, at pH = 5.0, FA-FRT-PFP disassembled and released PFP making it easier to undergo phase-transformation. Interestingly, when the LIFU irradiation time was extended to 4 min, FA-FRT-PFP at pH = 5.0 and 2.0 W/cm^2^ of LIFU had a weaker US signal intensity compared to that at a lower irradiation time, likely due to the LIFU at this condition is too strong that induce the bubbles rupture. These results demonstrated that the FA-FRT-PFP could release PFP, generating phase-transformations and explosion at 2.0 W/cm^2^ of LIFU irradiation at pH = 5.0. The results of US intensity are shown in Fig. 4b, c. Figure 4d–g shows the morphology of FA-FRT-PFP solutions after 3 min of LIFU irradiation (2.0 W/cm^2^) and at pH = 7.5 and 5.0. FA-FRT-PFP at pH = 5.0 and 2.0 W/cm^2^ LIFU generated large bubbles, further validating our findings.

### Cell Uptake

**Fig. 5:**
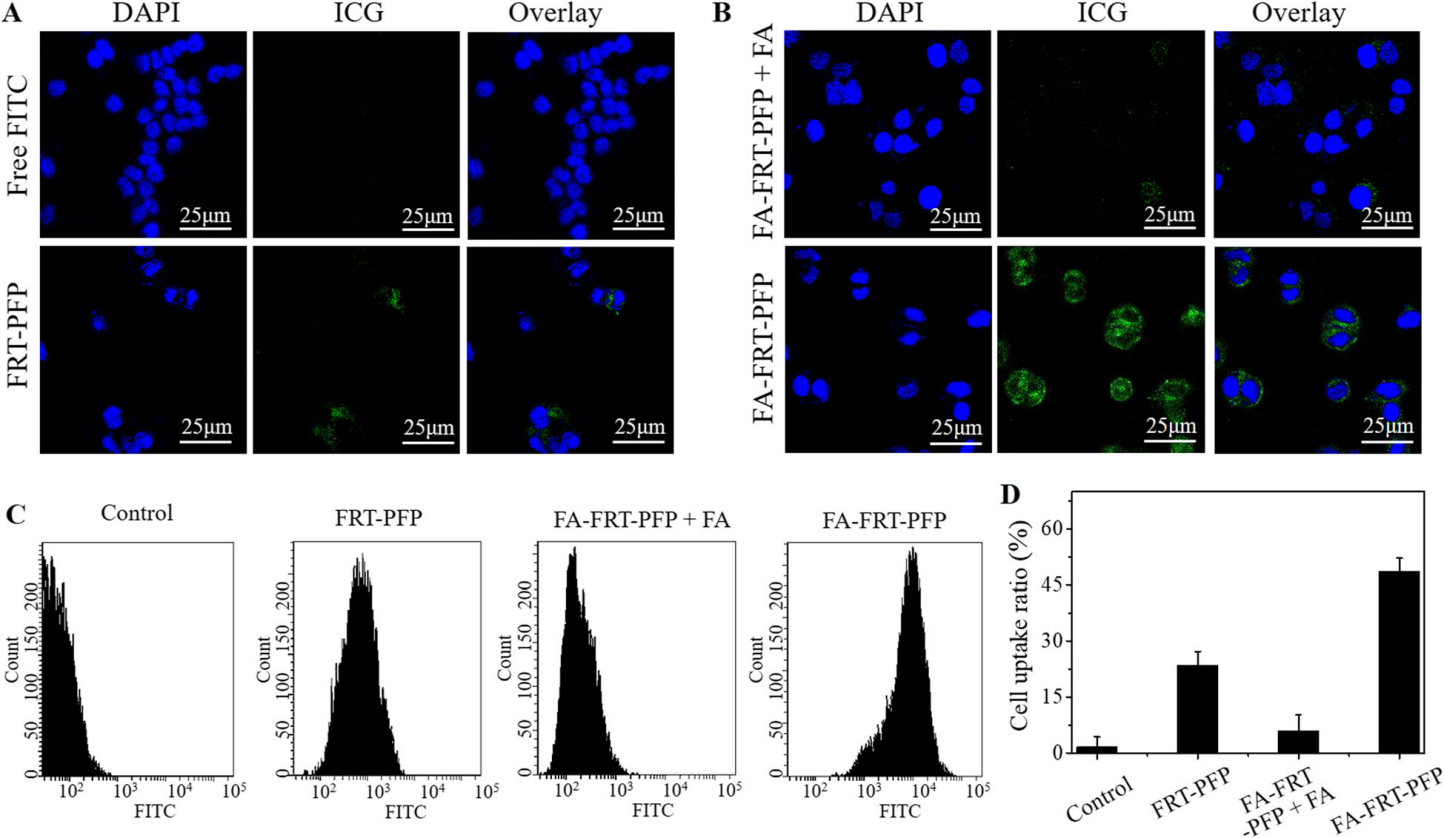
Cellular uptake. **a**, **b** The confocal fluorescence images of cells treated with free FITC and FITC-labeled FRT-PFP, FA-FRT-PFP + FA, and FA-FRT-PFP. Green and blue colors represented FITC and DAPI fluorescence, respectively. Scale bar = 25 μm. **c**, **d** The FITC fluorescence signal and the corresponding statistical data inside cells treated with free FITC and FITC-labeled FRT-PFP, FA-FRT-PFP + FA, and FA-FRT-PFP

Figure 5 shows the cellular uptake capacity of FA-FRT-PFP. Free FITC-treated cells showed negligible fluorescence but following labeling onto FA-FRT-PFP, a strong fluorescence signal was evident, indicating that FA-FRT-PFP has strong cellular uptake capacity. FRT-PFP-treated and FA-FRT-PFP-treated cells pre-incubated with FA showed weaker FITC fluorescence. This further demonstrated that FA enhances the active targeting of nanoparticles. In addition, FA-FRT-PFP showed a comparable uptake behavior to FCM (Fig. 5c, d). The uptake rates of FA-FRT-PFP were 46.5 ± 2.8%, which was significantly higher than that of free FITC (control), FRT-PFP and FA-FRT-PFP + FA. Furthermore, Additional file 1: Figure S2 shows the cellular uptake of nanoparticles in normal ovarian cells. There are no significant differences between FRT-PFP, FA-FRT-PFP + FA, and FA-FRT-PFP groups in cellular uptake. This is likely due to the normal ovarian cells (HUM-CELL-0088) have low FA receptor expression compared to SK-OV-3 cells.

### In Vitro Ultrasound Imaging

**Fig. 6:**
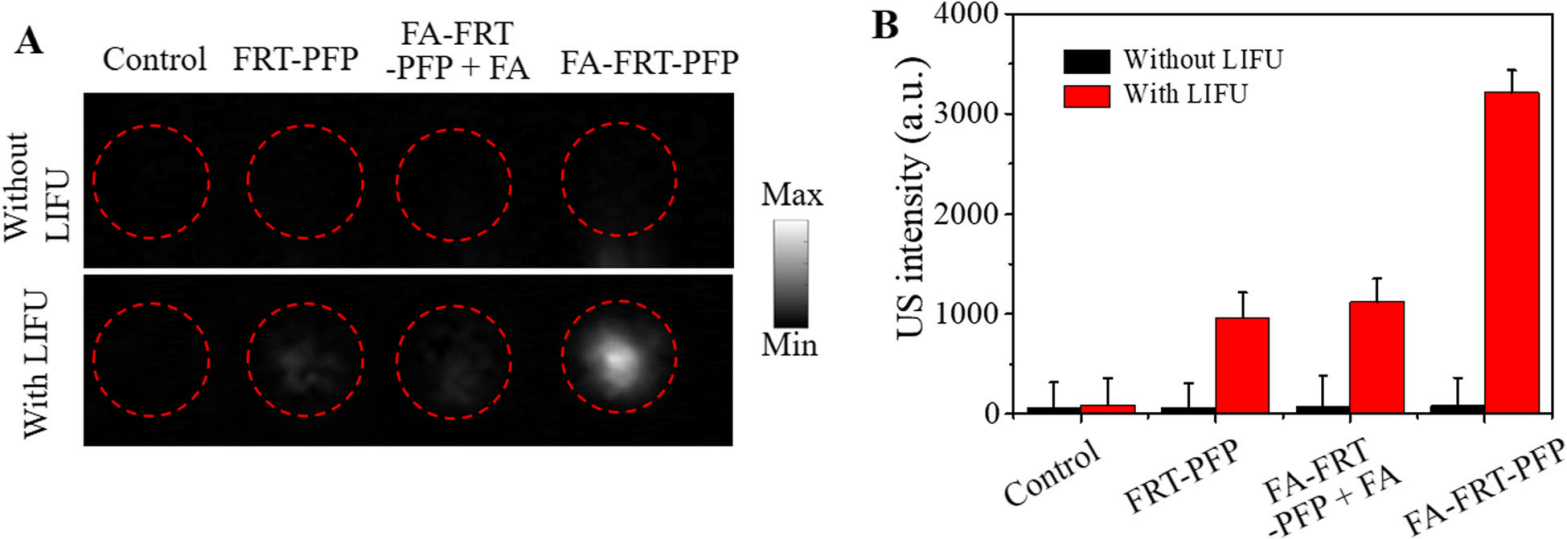
Ultrasound imaging in vitro. **a** The ultrasound images and the corresponding statistical data (**b**) of PBS (control), FRT-PFP, FA-FRT-PFP + FA, and FA-FRT-PFP-treated cells with or without LIFU irradiation (2.0 W/cm^2^, 3 min)

For confirmation that FA-FRT-PFP enhances ultrasound imaging by ADV in vitro, nanoparticles were incubated with the cells for 5 h and irradiated with or without LIFU (2.0 W/cm^2^). As shown in Fig. 6a in the absence of LIFU irradiation, cells in all tested groups showed no enhanced ultrasound signals while following LIFU irradiation, FA-FRT-PFP-treated cells showed high US intensities compared to control FRT-PFP and FA-FRT-PFP + FA groups, respectively. For ultrasound imaging, quantitative analysis of the grayscale values was calculated using ImageJ software. The results were consistence with the US imaging data (Fig. 6b) showing that the US intensity of FA-FRT-PFP treated cells were enhanced after irradiation by LIFU at 2.0 W/cm^2^ for 3 min. FA-FRT-PFP enters cells and in the acidic environment of lysosomes (pH = ~ 5.0) disassembled and released PFP into the cytoplasm [34, 35]. Following LIFU irradiation, the PFP easily generated phase-transformations from droplets to gas, which enhanced the US intensity.

### In Vitro Anticancer Efficacy and Cell Death Analysis

**Fig. 7:**
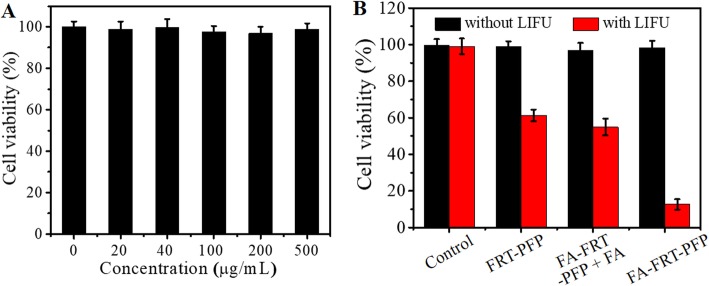
In vitro anticancer efficacy. **a** Cell viabilities of SK-OV-3 cells after 24 h incubation with different concentration of FA-FRT-PFP. **b** Cell viabilities of SK-OV-3 cells treated with 40 μg/mL of PBS (control), FRT-PFP, FA-FRT-PFP + FA, and FA-FRT-PFP combined with or without LIFU irradiation (2.0 W/cm^2^, 4 min) and further 21 h incubation

Figure 7a shows the cytotoxicity of FA-FRT-PFP at concentrations up to 0.5 mg/mL. In the absence of LIFU irradiation, FA-FRT-PFP showed no obvious suppression of viability in SK-OV-3 cells. CCK-8 assays were used to analyze the anticancer efficacy of FA-FRT-PFP combined with LIFU. As shown in Fig. 7b, in the absence of LIFU irradiation, FRT-PFP, FA-FRT-PFP + FA, and FA-FRT-PFP displayed no remarkable cytotoxicity compared to control groups. When combined with 4 min of LIFU irradiation, FA-FRT-PFP showed significant cytotoxicity which was higher than that of FRT-PFP, FA-FRT-PFP + FA, with peak cell inhibition rates approaching 90% at 40 μg/mL. This may be due to FA-FRT-PFP being more readily taken up into the cytoplasm and then lysosomes compared to FRT-PFP, FA-FRT-PFP + FA. In the acidic environment of lysosomes, FA-FRT-PFP released PFP into the cytoplasm. Under 4 min of LIFU irradiation, the released PFP gasified, causing cavitation and rupturing, generating a series of physical shock forces that significantly enhanced the killing effects of the tumors [36–38]. Moreover, Additional file 1: Figure S3 shows the cytotoxicity of nanoparticles in normal ovarian cells. There are no significant differences between FRT-PFP, FA-FRT-PFP + FA, and FA-FRT-PFP groups in cytotoxicity when they combined with LIFU. The result may be because HUM-CELL-0088 cells have low FA receptor expression that showed no FA targeting effect.

**Fig. 8:**
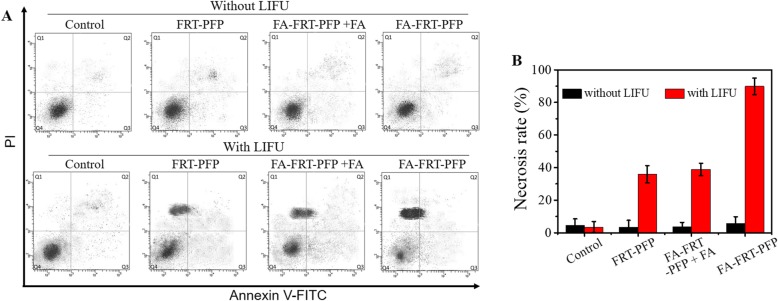
**a** Flow cytometry analysis and the corresponding statistical data of SK-OV-3 cells treated with 40 μg/mL of PBS (control), FRT-PFP, FA-FRT-PFP + FA, and FA-FRT-PFP combined with or without LIFU irradiation (2.0 W/cm^2^, 4 min) and further 23 h incubation

As shown in Fig. 8a, b, in the absence of LIFU irradiation, cells showed minimal cell death in all groups. Following 4 min of LIFU irradiation, FA-FRT-PFP-treated cells showed significant necrosis (~ 91.6%) compared to other groups due to the physical shock waves generated by LIFP-induced PFP cavitation or bubble blasting. Furthermore, Additional file 1: Figure S4 shows the TNF protein expression level of cells. Without LIFU, the cells hardly expressed TNF protein. While with LIFU, the cells in FA-FRT-PFP group have a high level of TNF expression, compared to that in FRT-PFP and FA-FRT-PFP + FA groups, indicating the TNF protein was a vital FA-FRT-PFP + LIFU-induced death-related protein [39]. Based on the excellent in vitro cancer therapy effect [40–42], FA-FRT-PFP is promising for future cancer therapy in vivo.

## Conclusion

In summary, we successfully developed FRT-based and PFP-loaded phase-transformation nanoparticles modified with an FA target molecule (FA-FRT-PFP). As a novel targeted US theranostic, FA-FRT-PFP had multiple advantages: (1) FA-FRT-PFP had a nanoscale particle size; (2) FRT was used as carrier, which could disassemble and reassemble to upload and release PFP droplets at acidic and neutral conditions, respectively; (3) under 3 min of LIFU assistance and acidic conditions, FA-FRT-PFP released PFP and generated phase-transformations through ADV, which could significantly increase its US contrast; (4) under 4 min of irradiation by LIFU and an acidic environment, the released PFP could easily induce an intracellular “explosion effect,” resulting in physical antitumor characteristics. These results demonstrate that FA-FRT-PFP with LIFU assistance provides a novel strategy for ultrasound molecular imaging and tumor therapy.

## Supplementary information

**Additional file 1: Figure S1**. The FT-IR spectrum of FA-FRT. **Figure S2**. The statistical data of FITC fluorescence signal inside HUM-CELL-0088 cells treated with free FITC and FITC labeled FRT-PFP, FA-FRT-PFP + FA and FA-FRT-PFP. **Figure S3**. Cell viabilities of HUM-CELL-0088 cells treated with 40 μg/ml of PBS (control), FRT-PFP, FA-FRT-PFP + FA and FA-FRT-PFP combined with or without LIFU irradiation (2.0 W/cm^2^, 4 min) and further 21 h incubation. **Figure S4**. The TNF protein expression level of cells treated with 40 μg/mL of PBS (control), FRT-PFP, FA-FRT-PFP + FA and FA-FRT-PFP combined with or without LIFU irradiation (2.0 W/cm^2^, 4 min) and further 21 h incubation.

## Data Availability

The conclusions made in this manuscript are based on the data which are all presented and shown in this paper.

## Abbreviations

ADV: Acoustic droplet vaporization
ARP: Acoustic response platform.
CCK-8: Cell counting kit-8
EDC: 1-Ethyl-3-(3-dimethylaminopropyl) carbodiimide
FA: Folic acid
FBS: Fetal bovine serum
FITC: Fluorescein isothiocyanate
FRT: Ferritin
LIFU: Low-intensity focused ultrasound
NHS: N-hydroxysuccinimide
PFP: Perfluoropentane
US: Ultrasound
