# Supplementary material for: pH- and acoustic-responsive platforms based on perfluoropentane-loaded protein nanoparticles for ovarian tumor-targeted ultrasound imaging and therapy
Source: Nanoscale Res Lett. 2020 Feb 3;15:31. doi: 10.1186/s11671-020-3252-z (PMC6997325; doi:10.1186/s11671-020-3252-z)
Supplement: Supplementary file 1 — Additional file 1: Figure S1. The FT-IR spectrum of FA-FRT. Figure S2. The statistical data of FITC fluorescence signal inside HUM-CELL-0088 cells treated with free FITC and FITC labeled FRT-PFP, FA-FRT-PFP + FA and FA-FRT-PFP. Figure S3. Cell viabilities of HUM-CELL-0088 cells treated with 40 μg/ml of PBS (control), FRT-PFP, FA-FRT-PFP + FA and FA-FRT-PFP combined with or without LIFU irradiation (2.0 W/cm2, 4 min) and further 21 h incubation. Figure S4. The TNF protein expression level of cells treated with 40 μg/mL of PBS (control), FRT-PFP, FA-FRT-PFP + FA and FA-FRT-PFP combined with or without LIFU irradiation (2.0 W/cm2, 4 min) and further 21 h incubation. [file 11671_2020_3252_MOESM1_ESM.docx]

**Supplementary Data**

**pH- and acoustic-responsive platforms based on perfluoropentane loaded protein nanoparticles for ovarian tumor targeted ultrasound imaging and therapy**

Jianping Li^1†^, Hong Ji^1†^, Yong Jing^2^, Shiguang Wang^2^*

^1^ Department of Geriatric Medicine, Sichuan Academy of Medical Sciences & Sichuan Provincial People’s Hospital, Chengdu, Sichuan 610041, China

^2^ Department of Imaging, Eastern Hospital of Sichuan Academy of Medical Sciences & Sichuan Provincial People’s Hospital, Chengdu, Sichuan 610000, China

*Corresponding author: Shiguang Wang

Email: [wangsg_im@hotmail.com](mailto:wangsg_im@hotmail.com)

Telephone: + 86-13684006886

Address: No. 585 Honghe North Road, Longquanyi District, Chengdu, Sichuan 610000, China

† These authors contribute equally to this work.


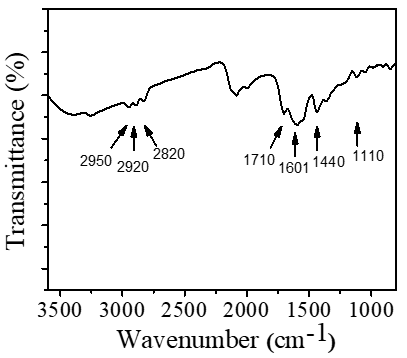


Figure S1. The FT-IR spectrum of FA-FRT.


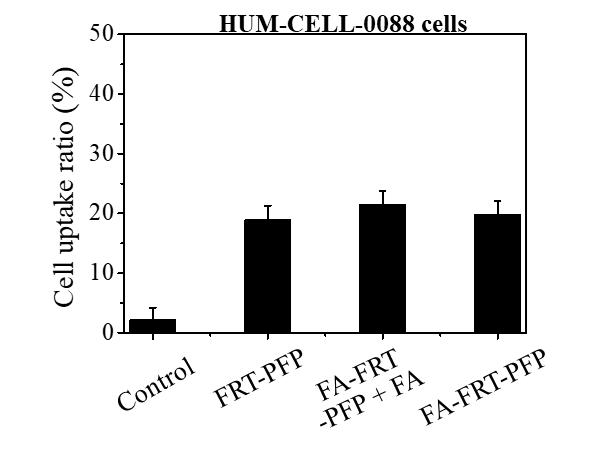


Figure S2. The statistical data of FITC fluorescence signal inside HUM-CELL-0088 cells treated with free FITC and FITC labeled FRT-PFP, FA-FRT-PFP + FA and FA-FRT-PFP.


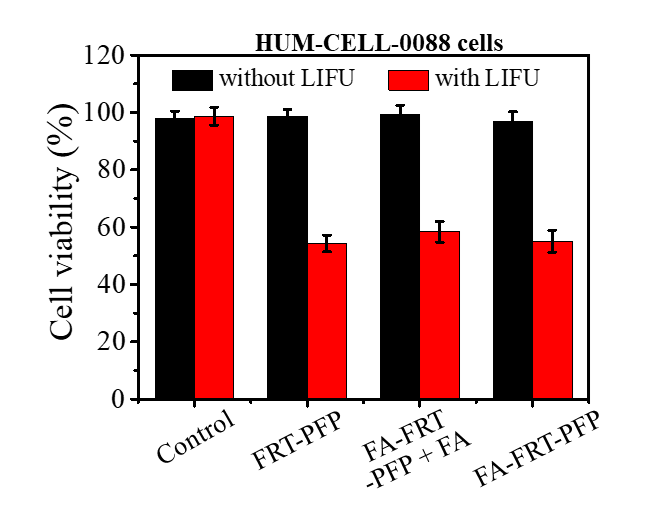


Figure S3. Cell viabilities of HUM-CELL-0088 cells treated with 40 μg/ml of PBS (control), FRT-PFP, FA-FRT-PFP + FA and FA-FRT-PFP combined with or without LIFU irradiation (2.0 W/cm^2^, 4 min) and further 21 h incubation.

**
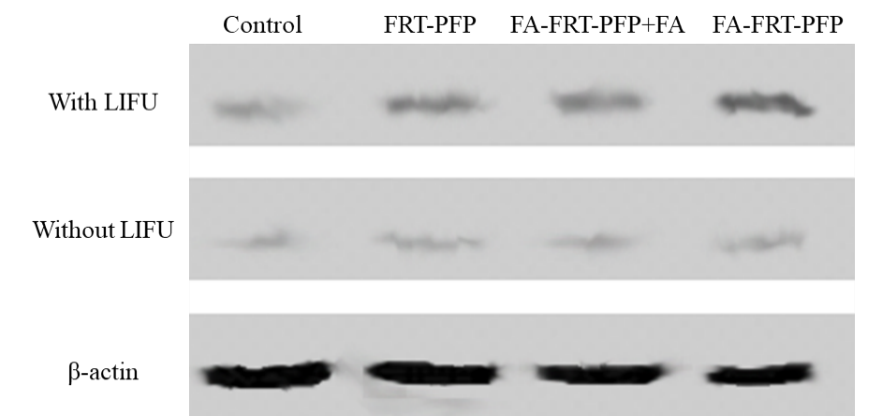
**

Figure S4. The TNF protein expression level of cells treated with 40 μg/mL of PBS (control), FRT-PFP, FA-FRT-PFP + FA and FA-FRT-PFP combined with or without LIFU irradiation (2.0 W/cm^2^, 4 min) and further 21 h incubation.
